# Metformin-induced chemosensitization to cisplatin depends on P53 status and is inhibited by Jarid1b overexpression in non-small cell lung cancer cells

**DOI:** 10.18632/aging.203528

**Published:** 2021-09-16

**Authors:** Tharcisio Citrangulo Tortelli, Rodrigo Esaki Tamura, Mara de Souza Junqueira, Janio da Silva Mororó, Silvina Odete Bustos, Renato Jose Mendonça Natalino, Shonagh Russell, Laurent Désaubry, Bryan Eric Strauss, Roger Chammas

**Affiliations:** 1 Centro de Investigação Translacional em Oncologia (LIM24), Departamento de Radiologia e Oncologia, Faculdade de Medicina da Universidade de São Paulo and Instituto do Câncer do Estado de São Paulo, São Paulo, SP 01246-000, Brazil; 2 Laboratory of Cancer Molecular Biology, Federal University of São Paulo, São Paulo, SP 04039-002, Brazil; 3 Department of Cancer Physiology, H. Lee Moffitt Cancer Center and Research Institute, Tampa, FL 33612, USA; 4 Laboratory of Regenerative Nanomedicine (RNM), INSERM U 1260, University of Strasbourg, CRBS, Strasbourg 67000, France

**Keywords:** metformin, cisplatin, Jarid1b, p53, NSCLC

## Abstract

Metformin has been tested as an anti-cancer therapy with potential to improve conventional chemotherapy. However, in some cases, metformin fails to sensitize tumors to chemotherapy. Here we test if the presence of P53 could predict the activity of metformin as an adjuvant for cisplatin-based therapy in non-small cell lung cancer (NSCLC). A549, HCC 827 (TP53 WT), H1299, and H358 (TP53 null) cell lines were used in this study. A549 cells were pre-treated with a sub-lethal dose of cisplatin to induce chemoresistance. The effects of metformin were tested both *in vitro* and *in vivo* and related to the ability of cells to accumulate Jarid1b, a histone demethylase involved in cisplatin resistance in different cancers. Metformin sensitized A549 and HCC 827 cells (but not H1299 and H358 cells) to cisplatin in a P53-dependent manner, changing its subcellular localization to the mitochondria. Treatment with a sub-lethal dose of cisplatin increased Jarid1b expression, yet downregulated P53 levels, protecting A549Res cells from metformin-induced chemosensitization to cisplatin and favored a glycolytic phenotype. Treatment with FL3, a synthetic flavagline, sensitized A549Res cells to cisplatin. In conclusion, metformin could potentially be used as an adjuvant for cisplatin-based therapy in NSCLC cells if wild type P53 is present.

## INTRODUCTION

Lung cancers are among the most lethal types of cancer worldwide. Approximately 80% of all lung cancers are Non-Small Cell Lung Carcinomas (NSCLC), which present an overall survival rate of 15%-35% for stage III-A and 5%-10% for stage III-B in the 5 years following diagnosis [1]. As platinum-based therapy for stage III NSCLC fails, novel approaches have been tested. Immunotherapeutic agents, like the immune checkpoint blocker anti-PD-1 monoclonal antibodies (Pembrolizumab or Nivolumab), present promising results, improving overall survival [2, 3]. However, the cost of this relatively new immunotherapy still limits access by most public health systems and patients around the world. Thus, new cost-affordable treatments are necessary.

Metformin, a biguanide derived from the plant *Galega officinalis*, has been used for many years to manage type-II diabetic patients [4, 5]. Metformin has become a candidate for repurposing to cancer therapy since the discovery that type-II diabetic patients treated with metformin have lower incidences of different types of cancer [6]. This effect has been studied successfully in many types of tumors [7, 8]. However, many other studies showed that metformin does not improve cancer treatment when in combination with chemotherapy [9, 10], demonstrating that more understanding is needed to determine when metformin can be used or not for cancer therapy.

Metformin accumulates in the mitochondrial matrix and blocks the complex-1 of the electron transport chain leading to a mitochondrial malfunction [11]. The resulting accumulation of ADP and AMP lead to the activation of the AMP-activated protein kinase (AMPK) that, among many other functions, inhibit AKT/mTOR pathway [12, 13]. Metformin also increases the glycolytic metabolism by enhancing glucose consumption, lactate production and by decreasing oxygen consumption [14]. Therefore, metformin could be used to chemosensitize subpopulations of tumor cells which depend preferentially on mitochondrial metabolism. This subpopulation is reprogrammed to escape the Warburg Effect, which relies on aerobic glycolysis. Mechanisms for metabolic reprogramming include the overexpression of the jumonji/ARID1 H3K4 histone demethylase Jarid1b/KDM5B/PLU-1, associated with the development of different types of tumors [15–17]. Jarid1b overexpressing cells are resistant to many types of chemotherapy (including cisplatin), display a mitochondrial-based primary metabolism and are responsible for tumor repopulation in melanoma [18]. Jarid1b overexpressing cells have a stem cell phenotype as demethylation of H3K4 blocks terminal differentiation of embryoid body and keeps high expression of stem cell markers like Oct-4 and Nanog [19].

Different responses of tumor cells to different treatments are related to circuitry rewiring of cancer gene signaling networks upon malignant transformation. For NSCLC, critical cancer driver gene networks involve *TP53* and EGFR-RAS-RAF-MEK-ERK pathways, which can be treated with EGFR inhibitors [20]. *TP53* is mutated in more than 50% of NSCLC. Patients harboring *TP53* mutations have a poorer prognosis than patients with wild type *TP53* [21]. Its major protein product is the multifunctional protein P53, which controls cell apoptosis at different levels. As a transcription factor, nuclear P53 induces the expression of apoptosis-related genes like *BAX*, *PUMA* and *NOXA* [22]. Cytoplasmic P53 can induce apoptosis when it is phosphorylated by AMPK- on serine 15. Ser^15^ –phosphorylated P53 translocate to mitochondria, where P53 can release BAX and BAK from BCL-XL, favoring the initiation of the apoptotic cascade [23].

FL3, a synthetic flavagline [24], has been reported as a stemness regulator by downregulating Oct4 in teratocarcinoma cell and selectively kills Oct-4 overexpressing cells and has little effect on normal cells. FL3-induced apoptosis depends on MAPK activation, as phosphorylation of p38 is necessary for FL3-iduced cell death [25]. Also, FL3 is a potent inhibitor of the EGFR-RAS-RAF axis, as it inhibits the activation of C-Raf by RAS through the inhibition of the interaction between C-Raf and the scaffold proteins prohibitin [26].

In the present study, we show that metformin chemosensitizes NSCLC cells to cisplatin in a P53-dependent manner. Sub-lethal treatment with cisplatin leads to Jarid1b overexpression and shifts the metabolism of A549 cells into glycolysis. Also, overexpression of Jarid1b leads to P53 downregulation, blocking the ability of metformin to sensitize to cisplatin-induced cell death. As Jarid1b overexpressing cells represent a chemoresistant population, FL3 could potentially be used to treat Jarid1b overexpressing cells. Therefore, P53-dependent pathways are critical for metformin-induced chemosensitization to cisplatin in NSCLC.

## RESULTS

### Metformin improves cisplatin-induced death in A549 and HCC 827 NSCLC cells in a P53 dependent manner

**Figure 1:**
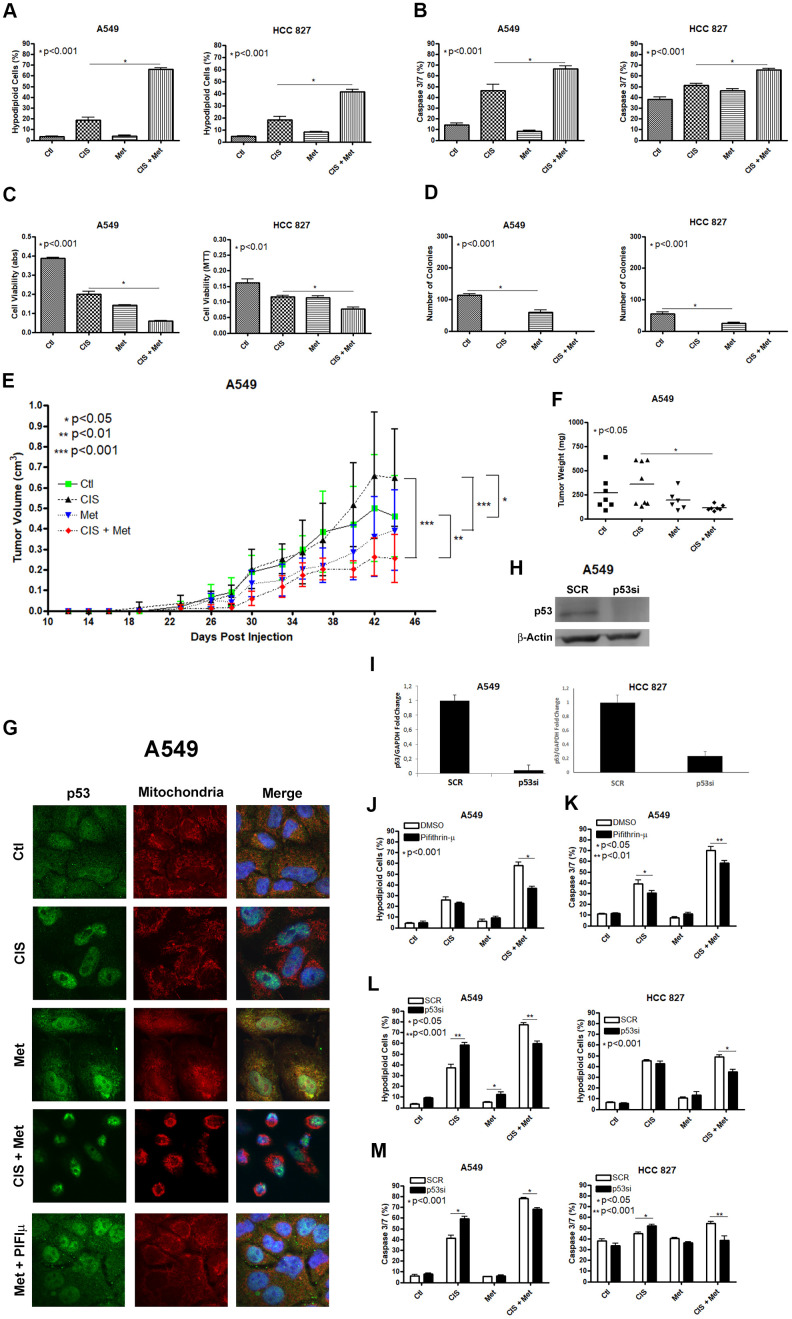
**Metformin improves cisplatin-induced death in A549 and HCC 827 NSCLC cells in a P53 dependent manner.** Combined treatment between cisplatin and metformin improves DNA fragmentation (p<0.001) (**A**), caspase 3 and 7 activation (p<0.001) (**B**) and cell viability assay through MTT (p<0.001 and p<0.01 respectively) (**C**), when compared to both treatments alone in A549 and HCC 827 cells. Metformin decreased the number of colonies and no colonies was observed after cisplatin treatment in A549 and HCC 827 cells (p<0.001 and p<0.01, respectively) (**D**). A549 cells injected in NOD/SCID mice also have a smaller volume (p<0.001) (**E**) and weight (p<0.05) (**F**) after combined treatment between cisplatin and metformin. Data represent the mean of three independent experiments. Metformin treatment translocate P53 to the mitochondria in A549 cells and this translocation is blocked by pifithrin-μ (**G**). P53 inhibition by pifithrin-μ protects A549 cells to the metformin induced chemosensitization to cisplatin by decreasing DNA fragmentation (p<0.001) (**J**) and caspase 3 and 7 activation (p<0.01) (**K**). *TP53* inhibition by siRNA (**H**, **I**) also protects A549 and HCC 827 cell from metformin-induced chemosensitization to cisplatin by decreasing DNA fragmentation (p<0.001) (**L**) and caspase 3 and 7 activation (p<0.001) (**M**). Data represent the mean of three independent experiments. A549 cells were treated with 10mM of metformin for 72 h and 25μM of cisplatin (with or without metformin) for another 72 h. HCC 827 cells were treated with 20mM of metformin for 72 h and 20μM of cisplatin (with or without metformin) for another 72 h.

We first checked the ability of metformin to chemosensitize the A549 and HCC 827 human non-small cell lung cancer cells lines to cisplatin. Combination of metformin with cisplatin synergistically induced a high chemosensitization to cisplatin evidenced through DNA fragmentation assay (Figure 1A), caspase 3 and 7 activation assay (Figure 1B) and through cell viability assay (Figure 1C) in A549 and HCC 827 cells. Metformin treatment reduced the ability of A549 and HCC 827 cells to produce colonies and this ability was completely abolished when these cells were treated with cisplatin (with or without metformin) (Figure 1D). A549 tumors in NOD-SCID mice treated with cisplatin plus metformin had the lowest tumor volume and weight, (Figure 1E, 1F and Supplementary Figure 1), yet cisplatin-only treated mice had the tumors with the highest volume, and this volume was not related to necrotic area (Supplementary Figure 2). As mentioned above, metformin-induced activation of AMPK leads to P53 phosphorylation, which promotes the induction of apoptosis. We first analyzed whether metformin treatment changes the subcellular localization of P53 to the mitochondria. Figure 1G shows that metformin increases P53 association with the mitochondria in A549 cells as seen by the yellowish color in the merged image. This accumulation can be partially blocked by pifithrin-μ, which specifically blocks P53 translocation to the mitochondria [27]. Also, cisplatin treatment accumulates p53 in the nucleus and no co-localization in the mitochondria was observed under cisplatin and metformin combination. To determine if the subcellular localization of P53 in the mitochondria is important for the metformin-induced chemosensitization to cisplatin we inhibited its localization with pifithrin-μ or inhibited its expression with siRNA. The inhibition of the subcellular localization of P53 by pifithrin-μ in A549 cells (Figure 1J, 1K) or the inhibition of its expression by siRNA in A549 and HCC 827 cells (Figure 1L, 1M), protected the A549 cells from metformin-induced chemosensitization to cisplatin after three days of combined treatment.

### Treatment with sub-lethal dose of cisplatin leads to Jarid1b overexpression and chemoresistance in A549 cells

**Figure 2:**
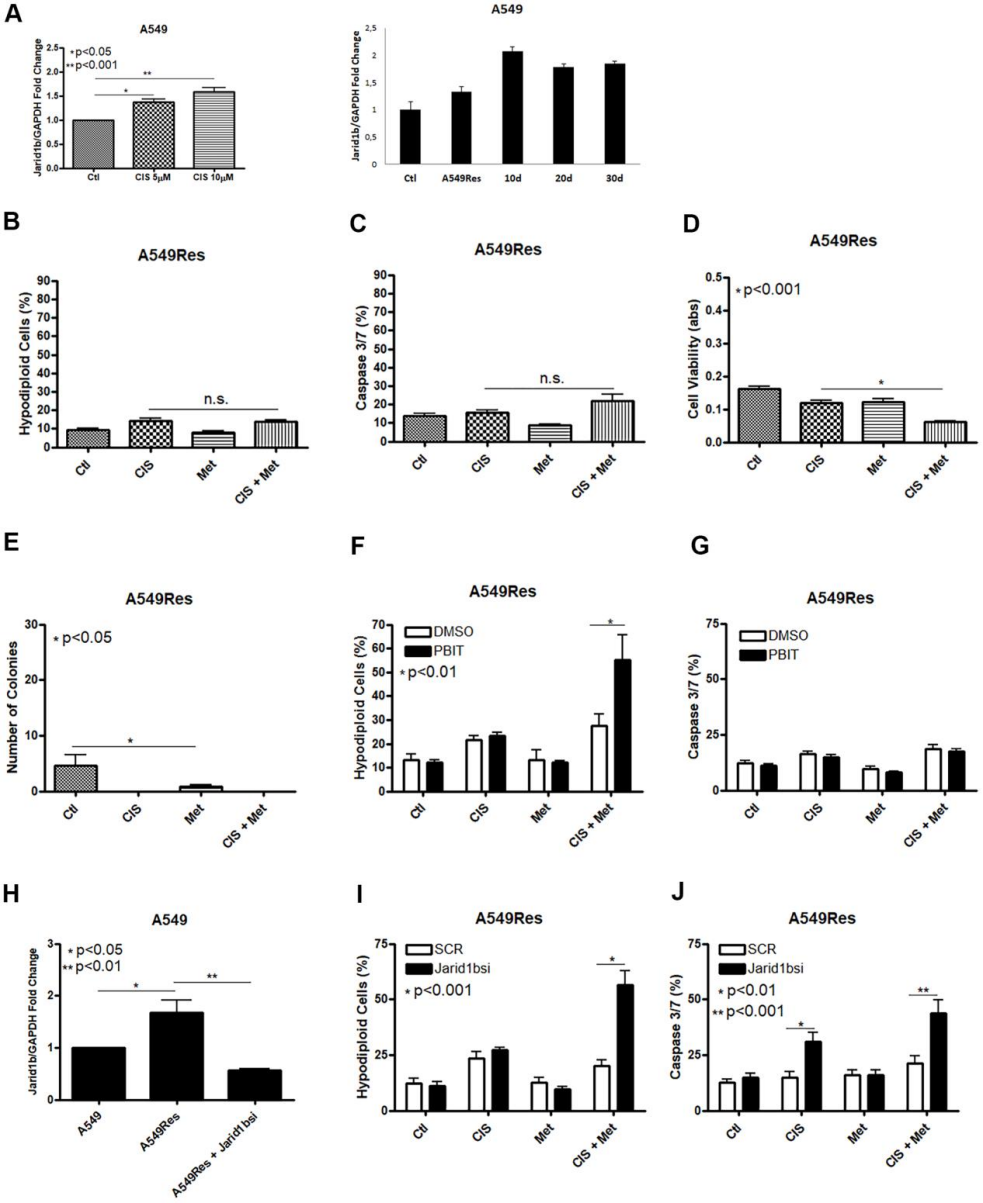
**Treatment with sub-lethal dose of cisplatin leads to Jarid1b overexpression and chemoresistance in A549 cells.** Sub-lethal treatment with cisplatin in A549 cells increases Jarid1b expression in a dose dependent manner and keeps overexpressed even after 30 days post-treatment (**A**). A549Res cells become resistant to the combined treatment between metformin and cisplatin as no improvement in DNA fragmentation (**B**), caspase 3 and 7 activation (**C**) can be seen. Only through cell viability assay showed some difference in the metformin and cisplatin combination (p<0.001) (**D**). Metformin decreased the number of colonies in A549Res cells (p<0.05), and no colonies was observed upon cisplatin treatment (**E**). Inhibition of Jarid1b by the pharmacological inhibitor PBIT restores the ability of metformin to chemosensitize to cisplatin as measured by DNA fragmentation assay (p<0.01) (**F**), but caspase 3 and 7 activation was not observed (**G**). However, inhibition of Jarid1b by siRNA (p<0.01) (**H**) restores the ability of metformin to chemosensitize to cisplatin through DNA fragmentation (p<0.001) (**I**) and caspase 3 and 7 activation assay (p<0.001) (**J**). Jarid1b inhibition by siRNA, MTT assay, DNA fragmentation and caspase 3 and 7 activation assay data represent the mean of three independent experiments. A549 cells were pre-treated with 10μM of cisplatin for 72 h to generate A549Res cells. After pre-treatment, A549Res cells were treated with 10mM of metformin for 72 h and 25μM of cisplatin (with or without metformin) for another 72 h.

Jarid1b overexpression is part of the survival response against different types of chemotherapy in melanoma cells [18]. The concept of using a sub-lethal dose of chemotherapy for a few days differs from the usual manner of emerging resistant cells *in vitro*, by treating them with a very low dose of chemotherapy for weeks. By giving a single sub-lethal dose, it is possible to compare the regular regiment of treatment, where in many cases, a few doses of chemotherapeutic agent are given to the patients, and due to the irregular distribution of chemotherapy in the tumor microenvironment, some regions of the tumor will receive a sub-lethal dose of the given chemotherapeutic agent and cell resistance could rise even with one shot [28, 29]. To check if A549 cells increase Jarid1b expression after cisplatin treatment we pre-treated these cells with a sub-lethal dose of cisplatin and observed an increase in Jarid1b mRNA expression in a dose-dependent manner that remains high for at least 30 days after the sub-lethal dose of cisplatin was completely removed and cells were kept in normal culture media (Figure 2A). In the following assays, A549Res cells were treated with a higher dose of cisplatin in combination with metformin. In the A549Res cells, metformin lost the ability to chemosensitize to cisplatin, as DNA fragmentation or caspase 3 and 7 activation under cisplatin and metformin treatment had the same levels, when compared to cisplatin alone (Figure 2B, 2C). While A549Res cells show lower MTT staining as compared to A549, Figure 2D shows that metformin and cisplatin do not change the viability levels with the same efficiency as seen in Figure 1C. Metformin also lowered the number of colonies in A549Res cells while no colonies were observed in A549Res cells after cisplatin treatment. However, the colonies formed after 30 days in A549Res cells have the same cell morphology as observed in A549 cells (Supplementary Figure 1F). Inhibition of Jarid1b by its pharmacological inhibitor PBIT [30] chemosensitized A549Res to the combination of metformin and cisplatin as seen in the DNA fragmentation assay, while caspase 3/7 activity was not elevated indicating that PBIT may activate a cell death pathway other than apoptosis (Figure 2F, 2G). To confirm that Jarid1b is related to chemoresistance in A549Res cells, a siRNA approach was employed. As expected, the siRNA to Jarid1b reduced its mRNA expression level (Figure 2H) and restored metformin-induced chemosensitization to cisplatin in the A549Res cells, as measured by DNA fragmentation (Figure 2I) and caspase 3 and 7 activation assays (Figure 2J).

### Sub-lethal dose of cisplatin inhibits P53 accumulation in a Jarid1b-dependent manner

**Figure 3:**
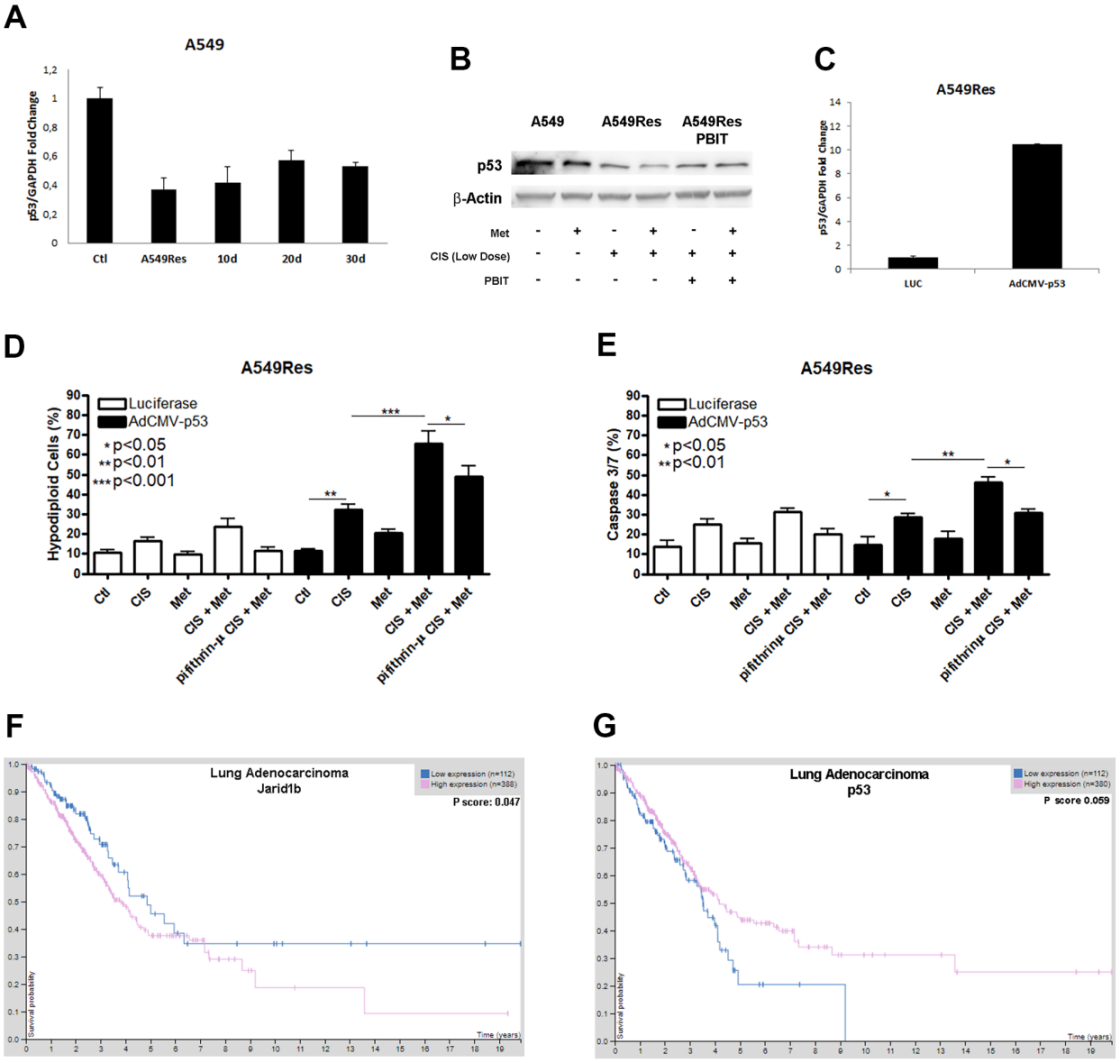
**Sub-lethal dose of cisplatin inhibits P53 accumulation in a Jarid1b-dependent manner.** Sub-lethal dose of cisplatin in the A549 cells downregulates *TP53* expression even after 30 days post-treatment (**A**). Western blot analysis shows that A549Res has lower expression of P53 compared to A549. Treatment with Jarid1b inhibitor PBIT avoid metformin-induced downregulation of P53 levels (**B**). Overexpression of *TP53* using AdCMVp53 expressing virus (**C**) restores metformin-induced chemosensitization to cisplatin on A549Res as seen by DNA fragmentation (p<0.001) (**D**) and caspase 3 and 7 activation assay (p<0.01) (**E**), while treatment with pifithrin-μ protect from metformin and cisplatin combination in the DNA fragmentation (p<0.05) and caspase 3 and 7 activation assay (p<0.05) (**D**, **E** respectively). High expression of Jarid1b (p<0.05) (**F**) or low expression of *TP53*, despite p=0.059 (**G**) indicate poor prognosis for patients with lung adenocarcinoma. DNA fragmentation assay data represents the mean of three independent experiments and caspase 3 and 7 activation assay represents the mean of two independent experiments.

As shown by other groups [31] and us, Jarid1b can inhibit P53 expression. Sub-lethal treatment with cisplatin inhibited P53 in A549Res cells at the mRNA and protein levels (Figure 3A, 3B). *TP53* downregulation, in Figure 3A, remained low in A549Res cells in the same manner as Jarid1b expression remained high in Figure 2A. The western blot shows that metformin treatment increased P53 inhibition in A549Res cells. Jarid1b inhibition with PBIT blocked the metformin-induced downregulation of P53. To confirm if the increase of P53 is sufficient to sensitize the A549Res cells to the combination of cisplatin and metformin, we transduced a virus expressing *TP53* in A549Res cells (Figure 3C). This higher level of *TP53* was enough to increase cell death in A549Res cells, as inhibiting P53 translocation to the mitochondria by pifithrin-μ protected from metformin-induced chemosensitization to cisplatin (Figure 3D, 3E). The same relation of high expression of Jarid1b (Figure 3F) or low expression of *TP53*, (despite p value, the curves clearly showed a difference between high and low expression of *TP53* in survival probability) (Figure 3G), as seen in Figures 2A, 3A for the A549 cells, leads to poor prognosis for patients. Also, KM Plot shows that high expression of Jarid1b indicates poor prognosis in stage 3 lung adenocarcinoma, while low expression indicates poor prognosis in stage 1 and 2 [32] (Supplementary Figure 3).

### Sub-lethal treatment with cisplatin enhances glycolysis in A549 cells

**Figure 4:**
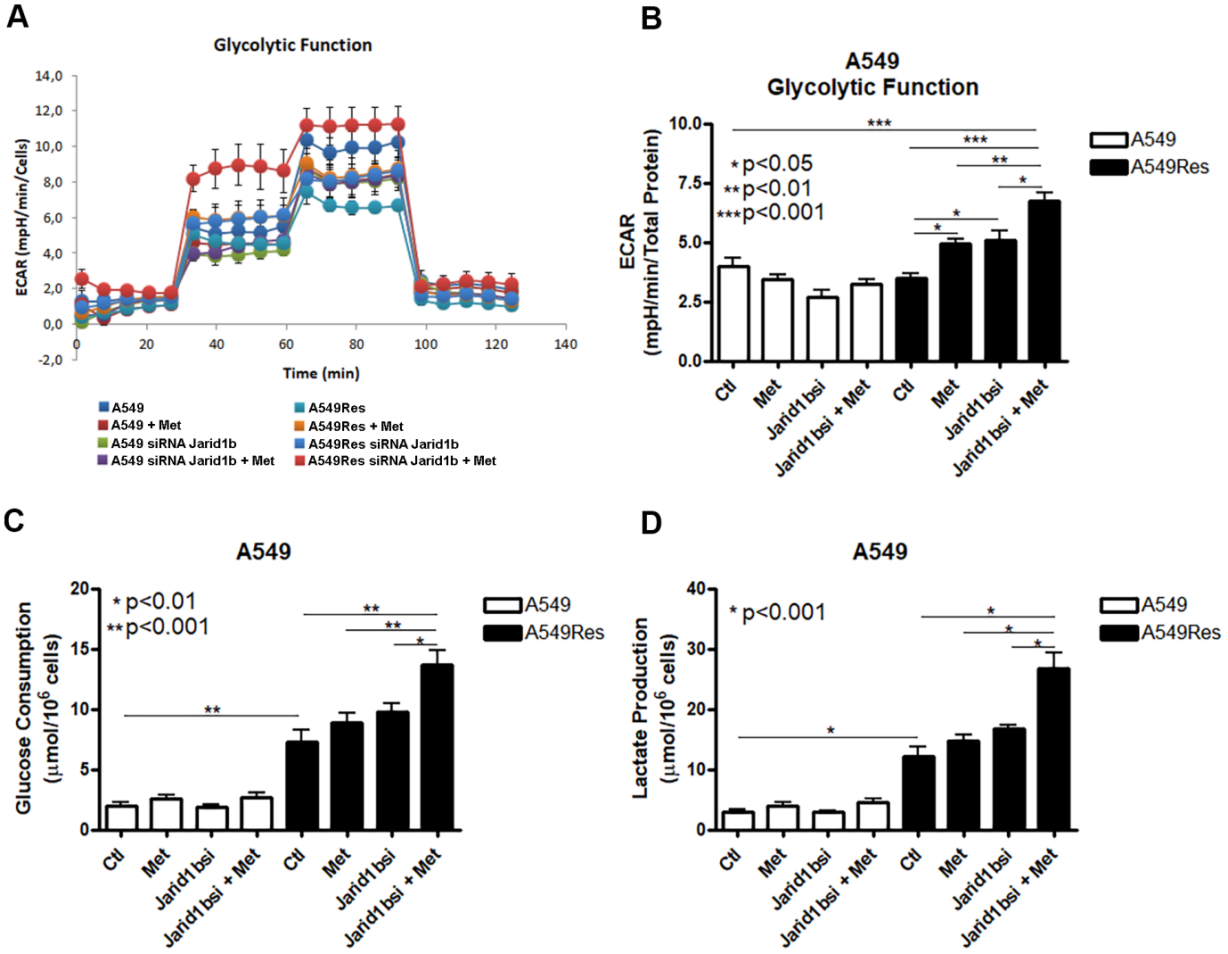
**Sub-lethal treatment with cisplatin enhances glycolysis in A549 cells.** A549 cells were treated with sub-lethal dose of cisplatin and treated with Jarid1b siRNA and metformin. Representative graph of glycolytic cell metabolism of A549 cells analyzed using the Seahorse XFe96 Analyzer (**A**). A549Res cells increase the extracellular acidification rate (ECAR) after metformin treatment (p<0.05) or Jarid1b inhibition by siRNA (p<0.05) or in the combination on both (p<0.001) (**B**). Glucose consumption (p<0.001) (**C**) or lactate production (p<0.001) (**D**) is increased after sub-lethal treatment with cisplatin for the generation of the A549Res cells. Data represent the mean of four independent experiments.

Tumor metabolism is a potential target for chemosensitization. Jarid1b overexpressing cells have a mitochondrial-based metabolism in melanomas where the inhibition of the mitochondrial function successfully restores the sensitization to chemotherapy [18]. As metformin fails to inhibit the mitochondria and chemosensitize the A549Res cells to cisplatin, we decided to analyze the metabolism of the A549Res cells. Surprisingly, the sub-lethal treatment with cisplatin in the A549Res cells increased the glycolytic metabolism, and not the OXPHOS metabolism, as expected due to Jarid1b overexpression. Figure 4A, 4B show that the extracellular acidification rate (ECAR) was increased in the A549Res cells upon metformin and/or inhibition of Jarid1b by siRNA. The increase in ECAR after Jarid1b inhibition shows the role of Jarid1b in promoting OXPHOS and that sub-lethal treatment with cisplatin impedes this function (Figure 4B). The glycolytic metabolism of the A549Res cells could be confirmed by two other parameters. Glucose consumption (Figure 4C) and lactate production (Figure 4D) were also increased upon sub-lethal treatment with cisplatin and Jarid1b inhibition by siRNA increased these parameters even more when cells were treated with metformin. Also, sub-lethal treatment with cisplatin decreased the oxygen consumption rate (OCR) and the mitochondrial activity in A549Res cells (Supplementary Figure 4).

### Metformin does not chemosensitize H1299 and H358 (P53 null) cells to cisplatin

**Figure 5:**
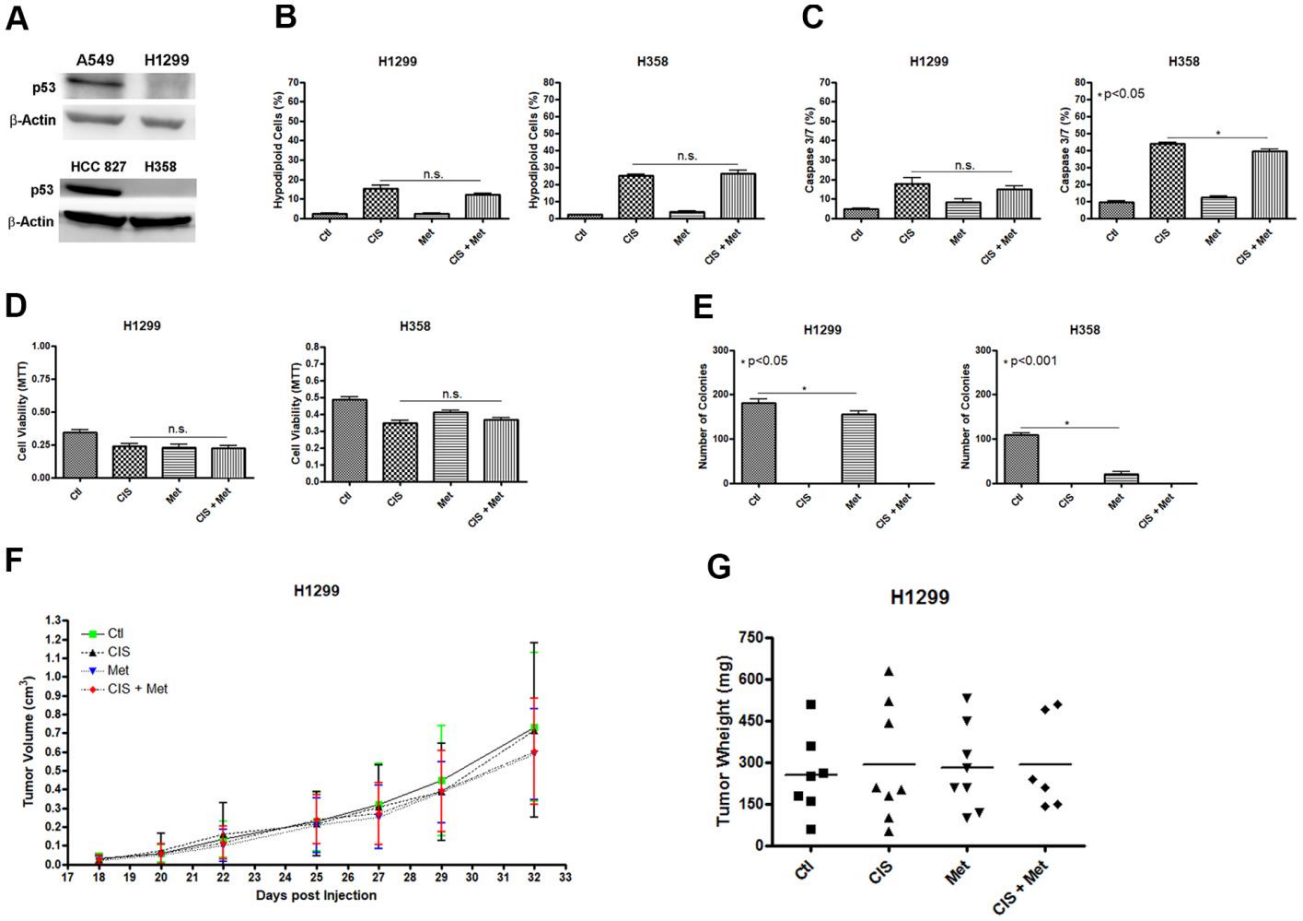
**Metformin does not chemosensitize H1299 and H358 (P53 null) cells to cisplatin.** The P53 null H1299 and H358 NSCLC cells (**A**) were not chemosensitized by combined treatment between cisplatin and metformin as it did not elevate DNA fragmentation (**B**), caspase 3 and 7 activation (**C**) or reduce cell viability as measured by MTT (**D**), when compared to either treatments alone. Metformin decreased the number of colonies and no colonies was observed after cisplatin treatment in H1299 and H358 cells (p<0.05 and p<0.001, respectively) (**E**). Combined treatment also did not decrease H1299 tumor growth (**F**) and weight in NOD/SCID mice (**G**). Data represent the mean of three independent experiments. Non-significance (n.s.). H1299 cells were treated with 2mM of metformin for 72 h and 12.5μM of cisplatin (with or without metformin) for another 72 h. H358 cells were treated with 20mM of metformin for 72 h and 20μM of cisplatin (with or without metformin) for another 72 h.

We analyzed whether the combination of cisplatin and metformin could sensitize another NSCLC cell line. We decided to use the human NSCLC cell line H1299 and H358 as these cells lack expression of P53 (Figure 5A). The results were the opposite as seen in the A549 cells, where P53 is present. In H1299 and H358, the combination of cisplatin and metformin did not increase cell death as no additional DNA fragmentation (Figure 5B) or no caspase 3 and 7 activation (Figure 5C) was observed. Also, no decrease in cell viability was seen after combined cisplatin and metformin treatment (Figure 5D). As observed in A549, A549Res and HCC 827 cells, metformin treatment decreased the ability of these cells to produce colonies and no colonies were observed when cisplatin was used as treatment (Figure 5E). To check if the H1299 cells could be chemosensitized to cisplatin by metformin *in vivo,* we used the same protocol previously applied to the A549 cells (Figure 1E, 1F). As expected, metformin did not chemosensitize the H1299 cells to cisplatin *in vivo* as no difference in tumor volume (Figure 5F) and weight (Figure 5G) was observed, despite a correlation between cisplatin and increased necrotic area in these tumors (Supplementary Figure 2). Differently than observed in A549 or HCC 827 and H1299 and H358 cells, the human ovary cancer cell lines SK-OV-3 and A2780 did not respond to cisplatin and metformin combination, independently of P53 status (Supplementary Figure 5).

### FL3 sensitizes A549Res but not H1299 cells to cisplatin

**Figure 6:**
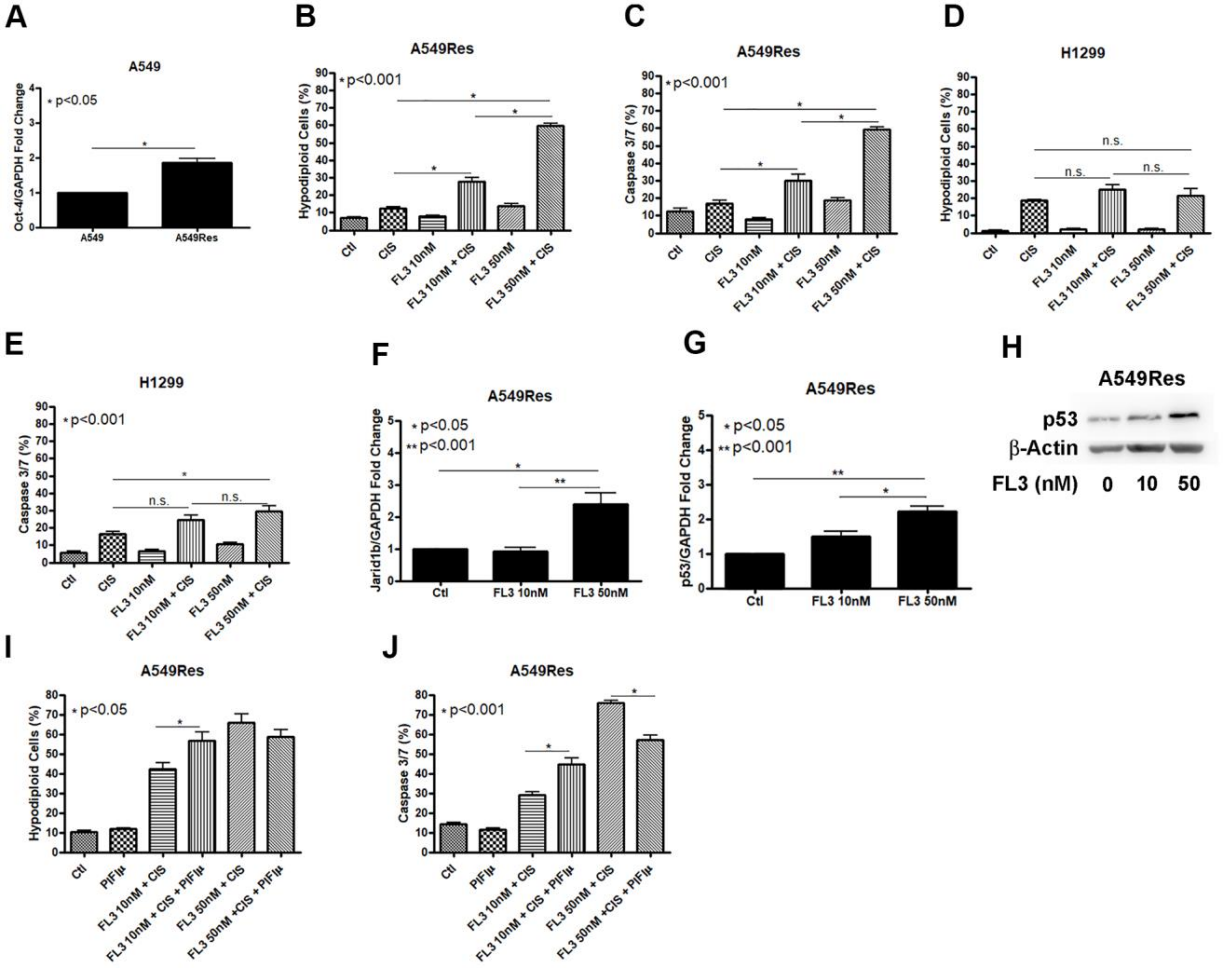
**FL3 sensitizes A549Res cells but not H1299 cells to cisplatin.** Sub-lethal treatment with cisplatin increased Oct-3 expression in A549Res cells (p<0.05) (**A**). The synthetic flavagline FL3 sensitized A549Res cells to cisplatin as measured by DNA fragmentation (p<0.001) (**B**) and caspase 3 and 7 activation (p<0.001) (**C**), Treatment with 50nM of FL3 combined with cisplatin did not increase DNA fragmentation on H1299, but increased caspase 3 and 7 activation, when compared to cisplatin alone (p<0.001) (**D**, **E**, respectively). In A549 cells, Jarid1b expression is increased upon FL3 treatment (p<0.05) (**F**). However, P53 expression is also increased upon FL3 treatment (p<0.001) (**G**, **H**). P53 inhibition by pifithrin-μ does not protects A549Res to cisplatin-induced cell death through DNA fragmentation assay but caspase 3 and 7 activation is lowered (p<0.001) (**I**, **J**, respectively). Data represent the mean of three independent experiments. FL3 treatment was kept during all experiment for H1299 and A549Res cells.

In an attempt to chemosensitize the A549Res cells to cisplatin, we used the synthetic flavagline FL3, as FL3 has been shown to induce the death of oct-4 high-expressing cells, like Jarid1b overexpressing cancer cells. We first analyzed if the sub-lethal treatment with cisplatin increase oct-4 levels in A549Res cells. Figure 6A shows that oct-4 expression was increased in A549Res cells. Cisplatin alone is enough to kill the A549Res cells after FL3 treatment in A549Res cells (Figure 6B, 6C) but is not enough to sensitize the H1299 cells (Figure 6D, 6E). In A549 cells, FL3 treatment increased Jarid1b expression in a dose-dependent manner (Figure 6F). However, FL3 treatment did not downregulate P53 and, instead, P53 levels were elevated, even though Jarid1b was overexpressed (Figure 6G, 6H). We asked if P53 localization in the mitochondria is necessary for FL3-induced chemosensitization to cisplatin. Figure 6I, 6J shows that pifithrin-μ did not reduce DNA fragmentation, yet did decrease the apoptotic pathway in A549Res cells, raising the possibility that an alternative cell death pathway compensated for the decrease in caspase 3/7 activity. Interestingly, the chemosensitization seen with high dose of FL3 treatment was diminished in the presence of metformin (Supplementary Figure 6).

## DISCUSSION

Non-small cell lung cancer is among the most lethal tumors and treatment frequently has a poor prognosis. A recent publication shows that metformin has no impact in platinum-based therapy for NSCLC [33]. Another publication shows that metformin enhances cisplatin-induced sensitivity to radiotherapy in two wild type P53 cell lines [34]. However, these two publications did not directly examine whether presence or not of P53 played a role in metformin-induced chemosensitization. Our work shows that the presence of P53 is necessary for the effectiveness of metformin-induced chemosensitization of cisplatin in NSCLC.

In this manuscript, we show that P53 is necessary for metformin-induced chemosensitization to cisplatin in NSCLC cells. In the A549 and HCC 827 cells, both wild type for P53, the combination of metformin and cisplatin increased the levels of DNA fragmentation and apoptosis activation, resulting in higher cell death, when compared to cisplatin and metformin treatment alone (Figure 1). In the H1299 and H358 cells, both P53 null, (Figure 5) and in the A549Res (Figure 2), no chemosensitization was seen. Therefore, using short-term treatment, as the doses used for cisplatin in all cells were too high to generate clones, P53 is necessary for metformin-induced chemosensitization to cisplatin in these cell lines. However, the same result was seen in the animal experiments for A549 and H1299 cells. We also show that Jarid1b overexpression is a cellular response to cisplatin, by downregulating P53 levels and protecting cells to the combined therapy. This *in vitro* and *in vivo* data is promising and warrants further investigation to work towards clinical studies.

The histone demethylase Jarid1b drives tumor metabolism towards OXPHOS and is a potential target for cancer therapy. Jarid1b overexpression is associated with poor prognosis in NSCLC [35] as seen by TCGA dataset (Figure 3F). Kaplan-Meier curve showed that Jarid1b is associated with poor prognosis in Stage 3 lung adenocarcinoma where the tumor is more resistant to treatment (Supplementary Figure 3C). In stage 1 and 2, Jarid1b low expression is associated with poor prognosis population (Supplementary Figure 3A, 3B) probably because Jarid1b can induce a slow-cycling and a long-term tumor maintaining population [18], making the tumors in these stages less aggressive. Biguanides like metformin and phenformin can target Jarid1b overexpressing cells, as these drugs can target the mitochondria, enhancing the effect of BRAF V600E inhibitors [36]. However, differently than expected, metformin works as a chemosensitizing agent to cisplatin in the A549 cells and not in the A549Res cells, where Jarid1b is overexpressed.

Roesch et al. showed that in melanomas, a biguanide like phenformin inhibited the mitochondria in Jarid1b overexpressing cells and restored the chemosensitivity of these cells [18]. In the A549 cells, metformin was not sufficient to restore the chemosensitivity of Jarid1b high expressing cells. The first reason could be because phenformin is a biguanide more powerful than metformin. However, phenformin increases lactic acidosis in patients, which can be potentially harmful. The second reason is because in the A549 cells, P53 is necessary for the ability of metformin to chemosensitize these cells to cisplatin. However, this mechanism still needs to be further explored as it seems to be tumor-context dependent as both the A2780 (P53 WT) and SK-OV-3 (P53 null) ovary cancer cell line does not respond to the combination of metformin and cisplatin (Supplementary Figure 5).

AMPK activation can phosphorylate and translocate P53 to the mitochondria and this translocation can be inhibited by pifithrin-μ [23, 37]. In a parallel study, metformin does not induce AMPK phosphorylation, using the same protocol used to generate the A549Res cells [38]. However, metformin treatment decreases mTOR activity in both A549 and A549Res cells, as expected. Thus, P53 translocate to the mitochondria in an AMPK-independent pathway in A549 cells. Figure 1 shows that P53 co-localization on the mitochondria is important for the chemosensitization of A549 cells as the inhibition of P53 translocation to the mitochondria by pifithrin-μ protects A549 cells from metformin-induced chemosensitization to cisplatin. It would be interesting to know whether *TP53* harboring any mutation will respond to cisplatin and metformin combination in NSCLC. One hypothesis is that even if a mutated *TP53* has lost its ability to transactivate target genes, it could chemosensitize NSCLC cells if the mutated P53 still has the ability associate with the mitochondria.

Tumor cell metabolism is a hallmark of cancer and is an important target for therapy. A subpopulation of tumor cells that uses oxidative phosphorylation as the main ATP source is resistant to chemotherapy and is generated by sub-lethal doses of many types of chemotherapy. One of these subpopulations has overexpression of the transcriptional coactivator peroxisome proliferator-activated receptor gamma coactivator-1 alpha (PGC-1alpha) which turns metabolism towards OXPHOS and protects cells from chemotherapy [39, 40] and is also overexpressed in A549Res cells (data not shown). To our surprise, sub-lethal treatment with cisplatin turns the metabolism towards glycolysis, even in the presence of Jarid1b. Jarid1b inhibition increases glycolysis after metformin treatment, showing that Jarid1b overexpression in A549Res cells still tries to hold the metabolism in the oxidative phosphorylation, but is overcome by the cisplatin treatment.

A549Res cells are resistant to the combination of cisplatin and metformin, indicating that a change in metabolism (i.e., increase in glycolysis) is not enough to their chemosensitization. In A549 cells, metformin does not increase glycolysis and, yet metformin is able to chemosensitize to cisplatin. Hence, metformin is chemosensitizing the A549 cells to cisplatin not by modulating the metabolism, but, at least in part, by using P53 as a chemosensitizing agent. Also, the increase in glycolysis in cisplatin-treated cells could explain the increase in tumor volume of A549 cells injected in NOD/SCID mice (Figure 1E), where tumors from the cisplatin-treated group had the highest volumes and weights (Figure 1E, 1F).

The rise of Jarid1b overexpressing cells upon cisplatin treatment seems to be transitory. Roesch et al. showed in melanoma model that the Jarid1b overexpressing cells lose Jarid1b expression spontaneously and become sensitive again to chemotherapy [18]. We observed that Jardi1b expression keeps high after 30 days of cisplatin treatment and the morphology of these cells changes to the non-resistant A549 morphology and cell starts to proliferate again (Supplementary Figure 1F). It seems that the cost of the resistance is the loss of proliferation in the A549 cells line. The genetic and/or epigenetic basis of this behavior still need to be understood.

In a parallel study [38], applying a proteomic approach to analyze the very same cell population groups studied here, A549Res cells showed that metformin decreased the expression of Lactate Dehydrogenase B (LDHB) and Succinate-CoA Ligase GDP-Forming Subunit Beta (SUCLG2), which can convert lactate into pyruvate and catalyze the conversion of succinyl-CoA to succinate, respectively [41, 42], on A549 cells. Nevertheless, metformin was not able to increase the lactate production in this population. Interesting, metformin treatment in A549 cells could decrease fatty acid oxidation enzymes like Electron Transfer Flavoprotein Subunit Alpha (ETFA) and Enoyl-CoA Hydratase 1 (ECH1), while increased the expression of Hydroxyacyl-CoA Dehydrogenase Trifunctional Multienzyme Complex Subunit Alpha (HADHA) in A549Res cells (data not shown). A549 cells are highly dependent on fatty acid oxidation, such dependence increases upon cisplatin treatment as observed in A549Res cells (Supplementary Figure 4C). Taken together, fatty acid oxidation could be a potential target to sensitize A549 cells to chemotherapy. Evidence in the literature showed that A549 cells in co-culture with differentiated 3T3-L1 adipocytes or harvested with its conditioned media increased cell proliferation, migration, invasion and fatty acid metabolism [43].

H1299 and H358 P53 null cells are resistant to the cisplatin and metformin combination (Figure 5). Despite H1299 cells being more sensitive to metformin and to cisplatin than the A549 cells (Supplementary Figure 7), and metformin decreased the number of colonies *in vitro*, metformin did not chemosensitize H1299 cells to cisplatin *in vivo*, as no difference in tumor growth and weight was seen using the same cisplatin and metformin dosage and the same treatment protocol as used in A549 cells, where the combination decreased tumor volume and weight (Figure 1E, 1F). This result shows that the lower dose of cisplatin and metformin used *in vitro* for the H1299 cells is not the reason that the combined treatment did not work for this cell line.

One of the ways that cisplatin can induce cell death is by enhancing reactive oxygen species (ROS) production in the mitochondria. Even though cisplatin treatment does not turn the metabolism into OXPHOS in A549Res cells through Jardi1b and PGC-1alpha (data not shown) overexpression, cisplatin-induced chemoresistance could still increase ROS protection as a chemoresistance mechanism. ROS production that could lead to cell death in A549 cells is related to the cisplatin treatment during the generation of the resistant A549Res cells and not to Jarid1b overexpression. No increment in ROS production is seen when comparing metformin-treated cells between A549Res and A549Res under PBIT treatment. Furthermore, pifithrin-μ, which protects A549 cells by blocking P53 translocation to the mitochondria, does not modify ROS production after metformin treatment in any condition (Supplementary Figure 8). Also, Jarid1b inhibition by PBIT does not increase ROS levels in A549Res after cisplatin and metformin combination (Supplementary Figure 8D). This evidence reinforces the idea that Jarid1b is protecting A549 cells through P53 downregulation and not by protecting them from ROS increase. Also, mitochondrial membrane potential and mitochondrial mass does not change under PBIT treatment (Supplementary Figure 8B, 8C).

In this work, we are showing that cisplatin treatment induces a chemoresistant subpopulation when the drug concentration is not strong enough to kill the tumor cells, that potentially will lead to treatment failure. It is urgent to find a way to block the rising of resistant cells after treatment. The stem cell behavior of Jarid1b overexpressing cells is related to the stem cell marker Oct4, as Jarid1b inhibition downregulates Oct4 [44]. The synthetic flavagline FL3 has been reported to selectively kill Oct4 overexpressing cells without affecting normal cells [25]. Therefore, FL3 could potentially kill cisplatin-induced chemoresistant cells in the tumor microenvironment, like Jarid1b overexpressing cells. FL3 was able to chemosensitize to cisplatin the A549Res cells but not H1299 cells (Figure 6), despite both cell lines expresses Oct4 [45]. Whether P53 is necessary or not for FL3 chemosensitization to cisplatin still need to be investigated. FL3 also prevent apoptosis in normal human skin cells, but not to malignant cells, through BAD activation [46] making it a good candidate for treatment where Jarid1b overexpressing subpopulation are present. This way, FL3 could be used in a treatment protocol, using cisplatin, where FL3 and metformin are not used at the same time as metformin protects from FL3-induced chemosensitization to cisplatin in A549Res cells when cells are treated with high doses of FL3 (Supplementary Figure 6).

## MATERIALS AND METHODS

### Cell culture, reagents, and primers for RT-PCR

A549, H1299 and HCC 827 NSCLC cells were purchased from Banco de Células do Rio de Janeiro. H358 cells were kindly provided by Dr. Daniela Basseres from Instituto de Química da USP. A549 cells were cultivated in Ham's F12 Nutrient Mixture media supplemented with 10% fetal bovine serum (FBS). H1299 NSCLC cells were cultivated in RPMI 1640 media supplemented with 10% FBS. HCC 827 and H358 were cultivated in RPMI 1640 media supplemented for 4000 mg/mL of glucose and 1mM of sodium pyruvate and supplemented with 10% fetal bovine serum. All experiments were made between passages 5 and 20 for A549 cells, between passages 28 to 40 for H1299 cells, between passages 35 to 50 for H358 and between passages 12 to 30 for HCC 827. All human cell lines were authenticated using Short Tandem Repeat (STR) profiling and pro filed within the last three years. All experiments were performed with mycoplasma-free cells. Cisplatin (cat: P4394), Metformin (cat: PHR1084), Pifithrin-μ (cat: P0122), PBIT (cat: sml1058), Thiazolyl Blue Tetrazolium Bromide (MTT) (cat: M2128) and Hoechst 33258 (cat: 94403) were purchased from Sigma-Aldrich. siRNA for *TP53* (cat: sc-29435) and Jarid1b (cat: sc-44522) were purchased from Santa Cruz Biotechnology. P53 antibody (cat: #9286) was purchased from Cell Signaling. Primers for Jarid1b (Forward: TGCTCCAGGTATCCCTTCCT Reverse: CCTCGGCAACAGTCATTCTTC); GAPDH (Forward: GGTGGTCTCCTCTGACTTCAACA Reverse: GGTGCTGTAGCCAAATTCGTTGT); *TP53* (Forward: CGCTTCGAGATGTTCCGAGA Reverse: CTTCAGGTGGCTGGAGTGAG); Oct-4 (Forward: TCTCCCATGCATTCAAACTGAG Reverse: CCTTTGTGTGTTCCCAATTCCTTC), CellEvent Caspase-3/7 Green Detection Reagent (cat: C10423), Opti-MEM (cat: 22600050), Mitotracker Red CMXRos (cat: M7512) and Alexa Fluor 488 (cat: A-11001) were purchased from ThermoScientific.

### Generation of A549 resistant cells (A549Res) and cell treatment

A549 cells were treated with sub-lethal dose of cisplatin (10 μM for 72 h) to generate cisplatin resistant cells (A549Res). A549 or A549Res cells were treated with metformin (10 mM for 72 h), prior to high dose of cisplatin (25 μM) and/or metformin for another 72 h. PBIT (20 μM) was added before the sub-lethal treatment with cisplatin and pifithrin-μ (15 μM) was added before metformin treatment and both were kept during all the experiment. H1299 cells were treated with 2 mM of metformin for 72 h and then, treated with 12.5 μM of cisplatin, combined or not with metformin for another 72 h. H358 and HCC 827 cells were treated with 20 mM of metformin for 72 h and then treated with 20 μM of cisplatin combined or not with metformin for another 72 h.

### Animal experiments

Seven weeks-old male NOD/SCID mice were divided in groups of eight animals with four animals per cage. In each cage, animals were randomized in different experimental group. Two million and a half cells/animal for the A549 cells and one million cells/animal for the H1299 cells were injected in the subcutaneous of the animals. Treatment started when palpable but not measurable tumors were detected. Commercial metformin, (Merck, lot number BR78614) was administered daily (350 mg/kg) diluted in 100 μL of regular mineral water through oral gavage for 14 days, starting one day before cisplatin treatment (four doses every 72 h, 2 mg/kg in PBS, intraperitoneally). All animals were sacrificed when the first tumor reached 1 cm^3^, for ethical reasons. Tumor volume was measured through the following equation: V=d^2^*D*0.52 (V: volume, d: minor diameter and D: major diameter).

### siRNA for *TP53* and Jarid1b

One hundred thousand cells/well of A549 or HCC 827 cells were plated on a 6 well plate. siRNA for Jarid1b (40 nM) or *TP53* (20 nM) was transfected with oligofectamine (8 μL/well) for 6h using Opti-Mem in the absence of fetal bovine serum. Oligofectamine and the siRNA were incubated alone in Opti-Mem media for 5 minutes alone and then were incubated together for 20 minutes for the oligofectamine-siRNA complex formation. After three washes with PBS, the Opti-Mem media containing the oligofectamine-siRNA complex was added to the 6 well plate (600 μL/well). After 6 hours, the Opti-Mem media containing the siRNA was replaced by the respective complete media. For a longer experiment using Jarid1b siRNA, a second inhibition was made after sub-lethal treatment with cisplatin.

### DNA fragmentation and caspase 3/7 assay

Thirty thousand cells/well were plated on a 12 well plate. For the DNA fragmentation assay, cells were fixed in 70% ethanol for 2 h at room temperature, washed once with PBS and incubated in 200 μL of propidium iodide (PI) solution (0.1% Triton X-100, 200 μg/ml of RNAse A and 20 μg/ml of PI) for 30 min at room temperature, protected from light. The hypodiploid content was used to estimate cells that were in cell death process. For Caspase 3 and 7 activation, cells were incubated with CellEvent Caspase-3/7 Green Detection Reagent (2 μM) in Ham's F12 Nutrient Mixture media with 10% FBS for the A549 cells or RPMI 1640 with 10% FBS for the H1299 cells at 37° C 5% CO_2_ incubator for 1h, protected from light. Caspase 3/7 positive cells was calculated through measurement of caspase 3/7 positive cells over caspase 3/7 positive and negative cells in flow cytometry.

### P53 adenoviral transduction

The non-replicating, serotype 5 adenoviral vectors AdCMVp53 and AdPGLuc have been described previously [47]. Virus purification in a gradient of iodixanol and titration using the Adeno-Xtm Rapid Titer Kit (Clontech) followed the procedures described in these previous publications. One hundred thousand A549 cells were plated in a 6 well plate and treated with 10 μM of cisplatin to generate A549Res cells. After 72 h the A549Res cells were transduced with the adenoviral vectors, AdPGLuc or AdCMVp53, using a multiplicity of infection (MOI) of 3 in Ham's F12 Nutrient Mixture culture medium with 2% fetal bovine serum. After 16 hours incubation at 37° C, cells were washed with PBS and cultivated in Ham’s F12 Nutrient Mixture medium containing 10% FBS. After 48 h, A549Res cells were treated accordingly to the protocol for chemosensitization to cisplatin by metformin.

### Protein extraction and western blot

Cells were trypsinized and centrifuged at 370 g for 2 min. Cell pellet was resuspended in RIPA buffer with protease inhibitor cocktail (Sigma cat: S8830) and left to stand at 4° C for 30 min. The homogenate was centrifuged at 4° C for 15 min at 13200 g and supernatant was collected. Protein content was measured with the BCA reagent kit (ThermoScientific cat: 23225). About 100 μg of proteins were separated on 10% polyacrylamide gel (0.375 M Tris, pH 8.8, 0.1% SDS, 10% acrylamide, 0.03% ammonium persulfate (APS), and 0.06% N,N,N′,N′- tetramethylethyilenediamine (TEMED)), and transferred to a hydrophobic polyvinylidene difluoride (PVDF) membrane. The nonspecific sites of the membrane were blocked with 5% fat-free milk in 0.1% PBS-Tween for 1 h at room temperature. PVDF membrane was incubated with the primary antibody overnight at 4° C. Membrane was washed three times with 5% fat-free milk in 0.1% PBS-Tween for 1 h at room temperature and the membrane was incubated with the secondary antibody for 1 hour at room temperature. The samples were visualized with the chemiluminescent substrate ECL (GE Healthcare).

### Cell viability through MTT

One two, or five thousand cells for the H1299, A549, A549Res cells, respectively, were plated on a 96 well plate. MTT solution (0.45 mg/mL final concentration) was added in culture media for 2 h in 37° C 5% CO_2_, protected from light. Cells were lysed in DMSO (150 μL/well), homogenized and the absorbance was read in 595 nm on a microplate reader.

### Clonogenic assay

Cells were treated according to their respective protocol. After treatment, 300 cells were plated in a 6 well plate and when colonies in the control group reached around 50 cells (7 days for H1299 cells; 9 days for A549, HCC 827 and H358 cells; 30 days for A549Res cells) cells were the washed with PBS and fixed with PBS/formaldehyde 4% for 15 minutes. Cells were washed again with PBS and incubated with crystal violet 0.1% for 10 minutes. After three washes with PBS, the plate was left for dry and then colonies were counted.

### Fluorescence microscopy

Ten thousand cells were plated onto a 24 well plate over a 30 mm coverslip where all treatments were made. For mitochondria labeling, Mitotracker Red CMXRos was diluted culture media (500 nM) and incubated at 37° C and 5% CO2 for 20 min. Cells were then fixed in 4% paraformaldehyde in PBS for 15 min and washed three times with PBS. 0.2% Triton X-100 in PBS was added for 5 min for cell permeabilization. Nonspecific sites were blocked in 5% PBS/BSA for 1 h. Primary antibody was incubated O.N. in 4° C. After three times washed in 1% PBS/BSA, secondary antibody conjugated with Alexa Fluor 488 (4 μg/mL) and Hoechst 33258 (0.5 mg/mL) were incubated for 1 h at room temperature in 5% PBS/BSA. After incubation, coverslips were mounted in a slide and cells were observed in the EVOS^©^ microscope.

### Glycolytic analysis, glucose consumption and lactate production

After respective treatment, A549’s culture media was removed, and cells were washed with PBS. New Ham's F12 Nutrient Mixture media, supplemented with 10% of FBS, was added in a volume of 1 ml. Cells were incubated in a 37° C and 5% CO_2_ for 1h. 200μL of the fresh A549 culture media was transferred to a 96 well plate in triplicate and the plate was transferred to the YSI 2950 Biochemistry Analyzer for glucose consumption and lactate production measurement Over 20000 cells of the remaining cells were plated in quintuplicate on the Seahorse XFe96 cell culture plate. Glycolysis Stress Test on the seahorse was made accordingly to user manual.

### TCGA data analysis

The survival curve comparing high and low expression of Jarid1b and *TP53* in lung adenocarcinoma was made in the Human Protein Atlas website (https://www.proteinatlas.org/), using the Cancer Genome Atlas (TCGA) RNA samples. All parameters were set to default.

### Statistical analysis

All statistical analysis was made in the GraphPad Prism software v4.03, using one or two-way ANOVA and Bonferroni for Post Test.

### Ethical approval

All procedures were in accordance with the guidelines of the Brazilian Council on Animal Care (COBEA) and approved by the Ethical Committee for Animal Research of School of Medicine (registry number: 100/16), University of São Paulo and the National Technical Commission on Biosafety (CTNBio), process number: 98509/2015.

## Supplementary Material

Supplementary Methods

Supplementary Figures
