# Supplementary material for: Metformin-induced chemosensitization to cisplatin depends on P53 status and is inhibited by Jarid1b overexpression in non-small cell lung cancer cells
Source: Aging (Albany NY). 2021 Sep 16;13(18):21914–40. doi: 10.18632/aging.203528 (PMC8507253; doi:10.18632/aging.203528)
Supplement: Supplementary Methods [file aging-13-203528-s001.pdf]

SUPPLEMENTARY METHODS

Clonogenic assay

Cells were treated according to their respective protocol. After treatment, 300 cells were plated in a 6 well plate and when colonies in the control group reached around 50 cells (7 days for H1299 cells; 9 days for A549, HCC 827 and H358 cells; 30 days for A549Res cells) cells were the washed with PBS and fixed with PBS/formaldehyde 4% for 15 minutes. Cells were washed again with PBS and incubated with crystal violet 0.1% for 10 minutes. After three washes with PBS, the plate was left for dry and then colonies were counted.

Oxygen consumption rate and fatty acid dependence analysis on seahorse XFe96

Twenty thousand cells were plated in quintuplicate for each experimental condition on Seahorse XFe96 Cell Culture Microplates one day before the experiment. Seahorse XF Cell Mito Stress Test and dependency test of Seahorse XF Mito Fuel Flex Test were done according to user manual.

Detection of superoxide anions by MitoSOX and measurement of the mitochondrial membrane potential (Δψm) and mitochondrial mass

The fluorescent probes were purchased from Thermo Fisher, Waltham, MA USA. At the end of the experimental treatments, cells were loaded with 3 μM of MitoSOX for 30 min at 37° C in HBSS solution (Gibco). In order to analyze the mitochondrial potential and mitochondrial mass, cells were incubated with TMRE (100 nM) for 20 min at 37° C and with Mitotracker green (150 nM) for 30 min at 37° C in HBSS, respectively. The evaluation of these three probes was performed by flow cytometry. For cytometry experiments the cells were counted and normalized before the probe incubation and analyzed using the geometric mean of the median fluorescence intensity with FlowJo software (Tree Star Inc., Ashland, OR, USA).

Cell culture and reagents

A2780 human ovary carcinoma cells were purchased from Banco de Células do Rio de Janeiro and SK-OV-3 human ovary carcinoma cells were kindly provided by Dr. Érico Tosoni Costa from Hospital Sírio-Libanês. All human cell lines were authenticated using Short Tandem Repeat (STR) profiling and pro filed within the last three years. All experiments were performed with mycoplasma-free cells. A2780 were cultivated in RPMI-1640 medium containing 4500 mg/L of glucose and supplemented with 10% FBS. SK-OV-3 human ovary adenocarcinoma cells were cultivated in McCoy's 5a Medium Modified media and supplemented with 10% FBS. All experiments were made from passage 6 to 17 for A2780 cells and from passage 16 to 25 for SK-OV-3 cells. Cisplatin (cat: P4394) and Metformin (cat: PHR1084), were purchased from Sigma-Aldrich. P53 antibody (cat: #9286) was purchased from Cell Signaling.

Treatment with cisplatin and metformin on the SK-OV-3 and A2780 cells

Forty thousand SK-OV-3 cells (human ovary adenocarcinoma cell line) were plated on a 12 well plate and treated with 20 mM of metformin for 72 h prior to the treatment with cisplatin (4 μM), combined or not with metformin, for another 72 h. Five thousand A2780 cells (human ovary carcinoma cell line) were plated in a 12 well plate and treated with 5 mM of metformin for 72 h prior to the treatment with cisplatin (2.5 μM), combined or not with metformin, for another 72 h.

Kaplan-Meier plotter of lung adenocarcinoma and expression of Jarid1b in different types of tumors

Overview expression of Jarid1b in different tumors was done in The Human Protein Atlas website (https://www.proteinatlas.org/), using the The Cancer Genome Atlas (TCGA) RNA samples. All parameters were set to default. Kaplan-Meier plots for the analysis of stages 1, 2 and 3 of lung adenocarcinoma were created at the http://kmplot.com/analysis/index.php?p=service&cancer=lung website. All parameters were set to default, using all available cohorts. Green JetSet color was chosen for the best probe set for KDM5B (Jarid1b) gene.
