# Supplementary material for: Metformin-induced chemosensitization to cisplatin depends on P53 status and is inhibited by Jarid1b overexpression in non-small cell lung cancer cells
Source: Aging (Albany NY). 2021 Sep 16;13(18):21914–40. doi: 10.18632/aging.203528 (PMC8507253; doi:10.18632/aging.203528)
Supplement: Supplementary Figures [file aging-13-203528-s002.pdf]

SUPPLEMENTARY FIGURES

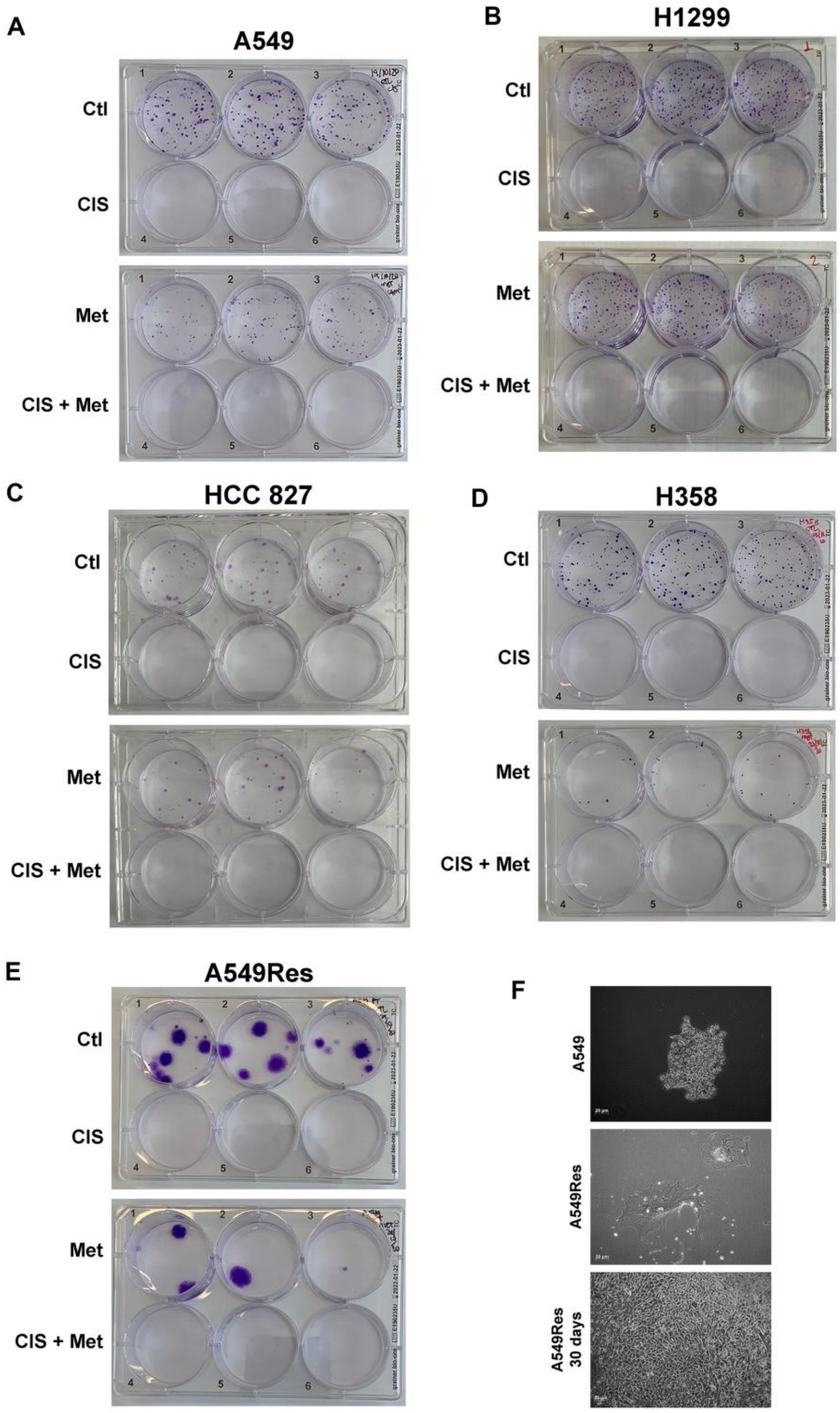

Supplementary Figure 1. Clonogenic assay in A549, H1299, HCC 827, H358 and A549Res cells. Cells were treated as described in methods section. After treatment, cells were detached from plate and 300 cells were plated in 6 well plate in triplicate. Colonies were then counted for A549 cells (**A**), H1299 cells (**B**), HCC 827 cells (**C**), H358 (**D**) and A549Res (**E**). In (**F**) a picture was taken to document the difference in morphology of the A549Res cells after 30 days when compared to A549 cells. Picture was taken from a Control group well in A549Res plate. All plates are representative of three independent experiments.

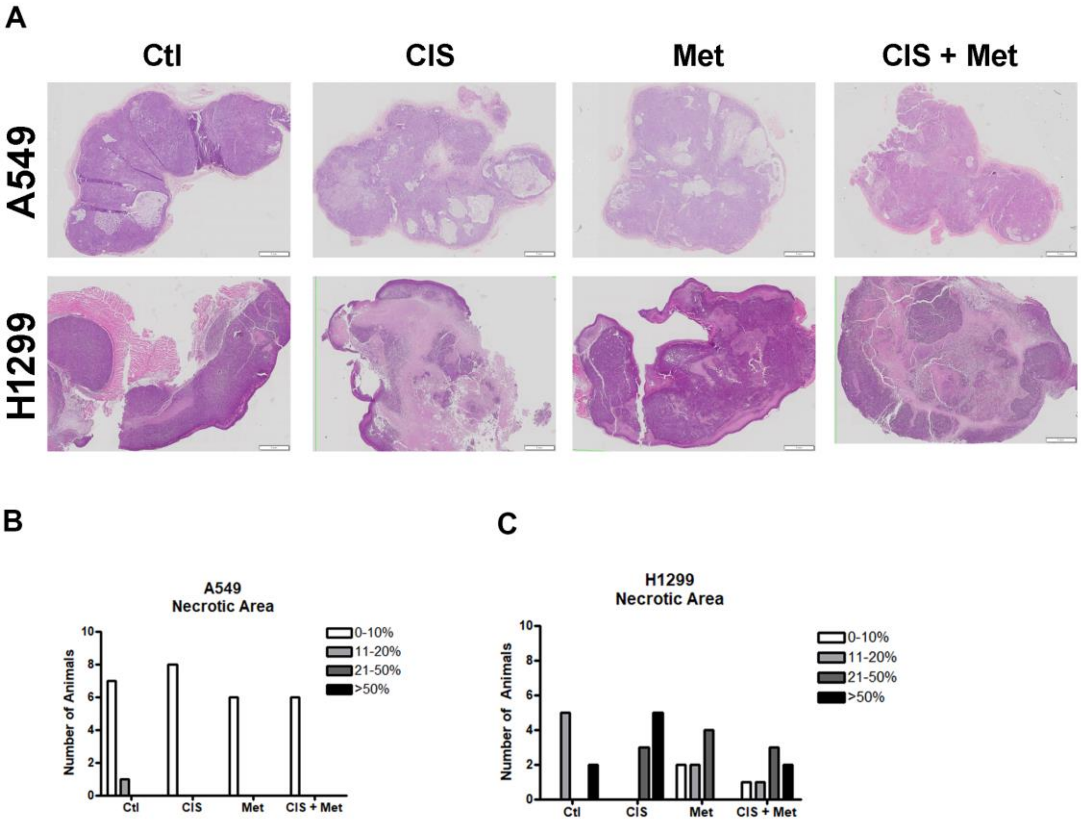

Supplementary Figure 2. Necrotic area in A549 and H1299 tumors injected in NOD/SCID mice. Histological section of A549 or H1299 derived tumors, stained with hematoxylin and eosin, after metformin, cisplatin and the combination of metformin and cisplatin treatment in NOD/SCID mice (**A**). Quantification of the necrotic area shows that in A549 derived-tumors, cisplatin does not increase the necrotic area (**B**). However, in H1299 derived-tumors, cisplatin increases the necrotic area when used alone in NOD/SCID mice and metformin decreases the necrotic area when combined with cisplatin (**C**).

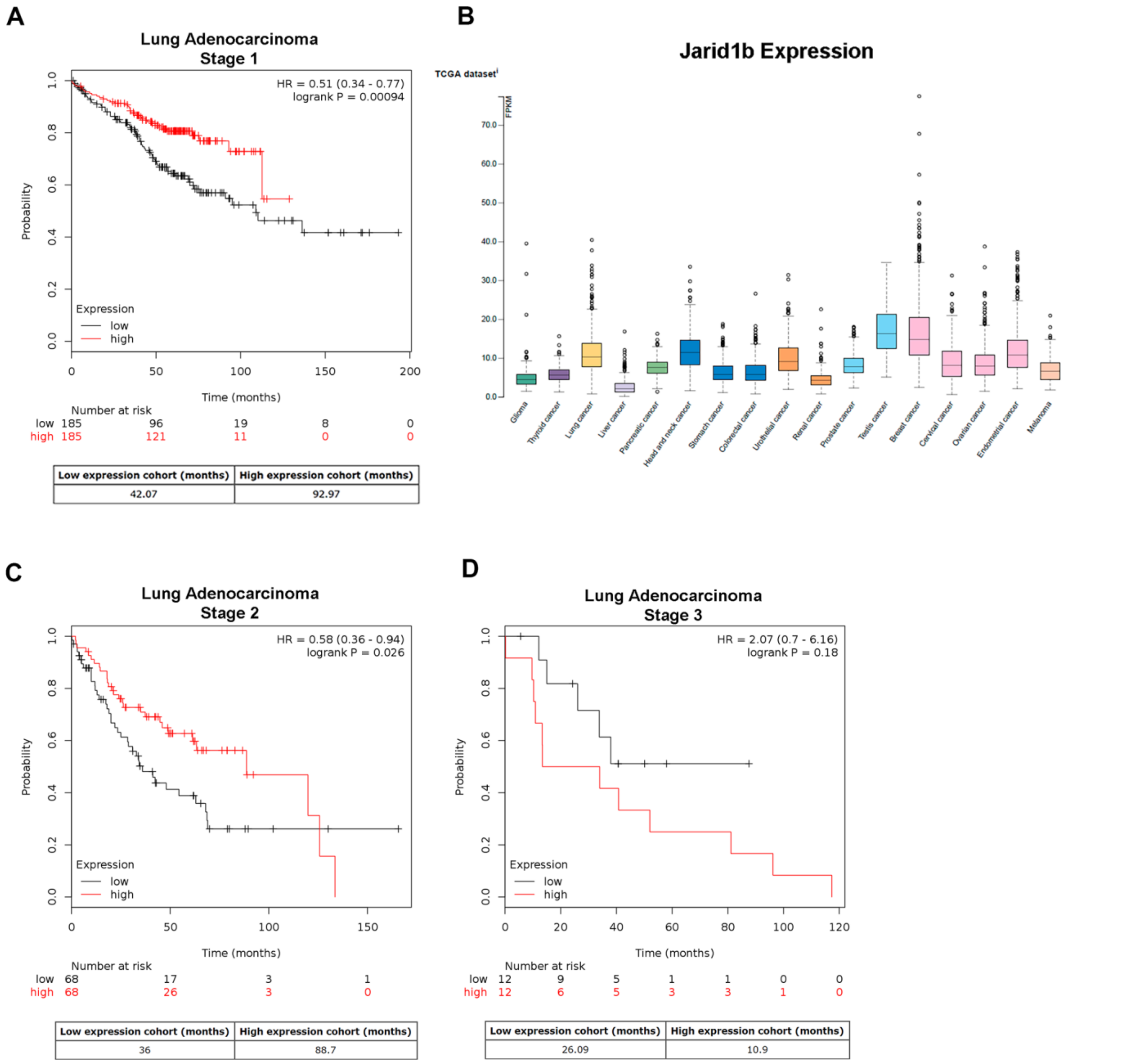

Supplementary Figure 3. Kaplan-Meier plotter of Jarid1b expression in lung adenocarcinoma. Jarid1b is highly expressed among different types of cancer (**A**). The Kaplan-Meier plotter shows that overexpression of Jarid1b in Lung Adenocarcinoma leads to a good prognosis in Stage 1 (p<0,001) (**B**), Stage 2 (p<0.05) (**C**) and has a tendency of poor prognosis in Stage 3 (p=0.18) due to the small number of patients (**D**).

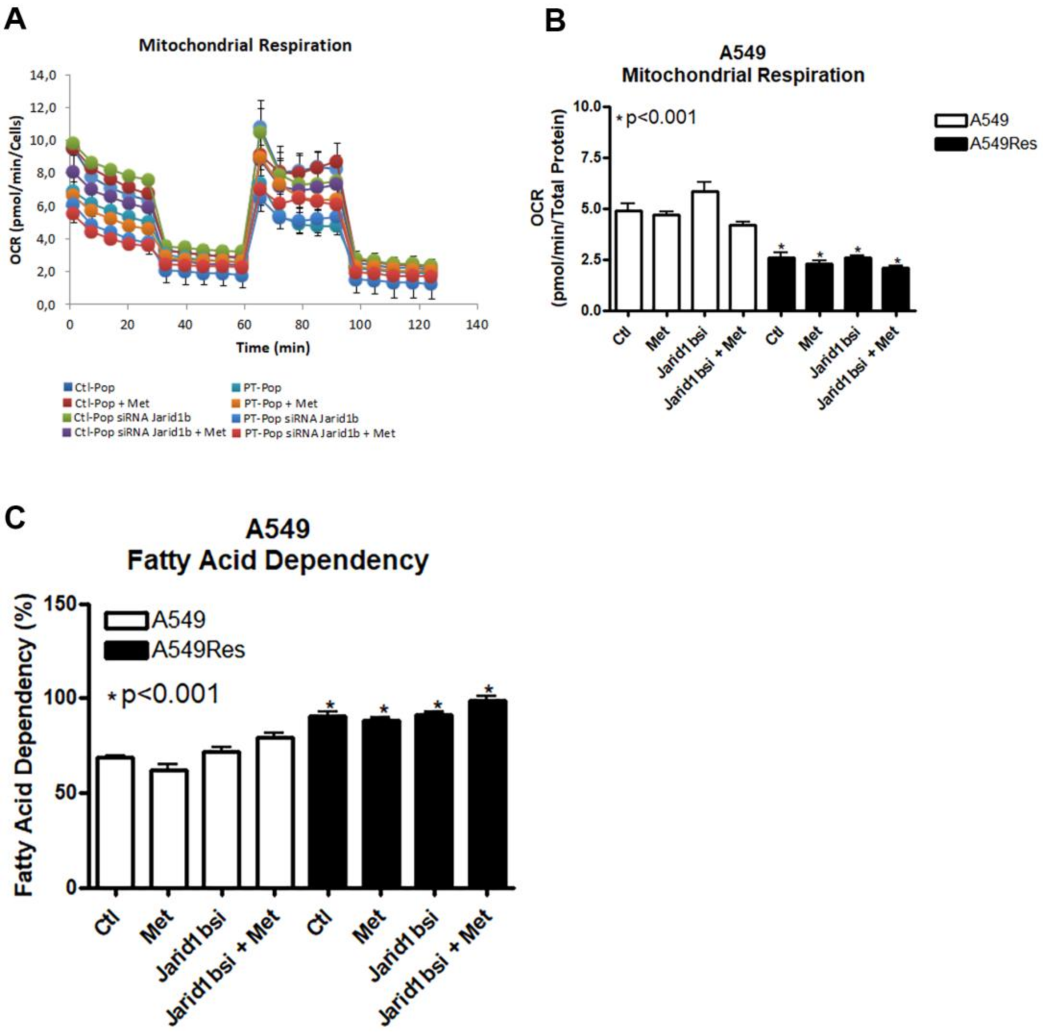

Supplementary Figure 4. Oxygen consumption rate (OCR) and fatty acid dependency in A549 cells. OCR in A549 cells or in A549Res cells were analyzed using the Seahorse XFe96. Cells were treated with metformin under Jarid1b inhibition by siRNA. Representative graph of four independent experiments from the measurement in A549 cells (**A**). Quantification of OCR in A549 and A549Res cells indicates that in the A549Res cells (when compared to the A549 Ctl group), OCR is decreased and metformin treatment and/or Jarid1b inhibition by siRNA did not increase its level ($p<0.001$) (**B**). A549 cells are highly dependent on fatty acid metabolism and this dependency increases after cisplatin treatment ($p<0.001$) (**C**). Sub-lethal treatment with cisplatin, metformin treatment and Jarid1b inhibition by siRNA was made according to materials and methods. OCR data represent the mean of four independent experiments and fatty acid dependence data represent the mean of three independent experiments.

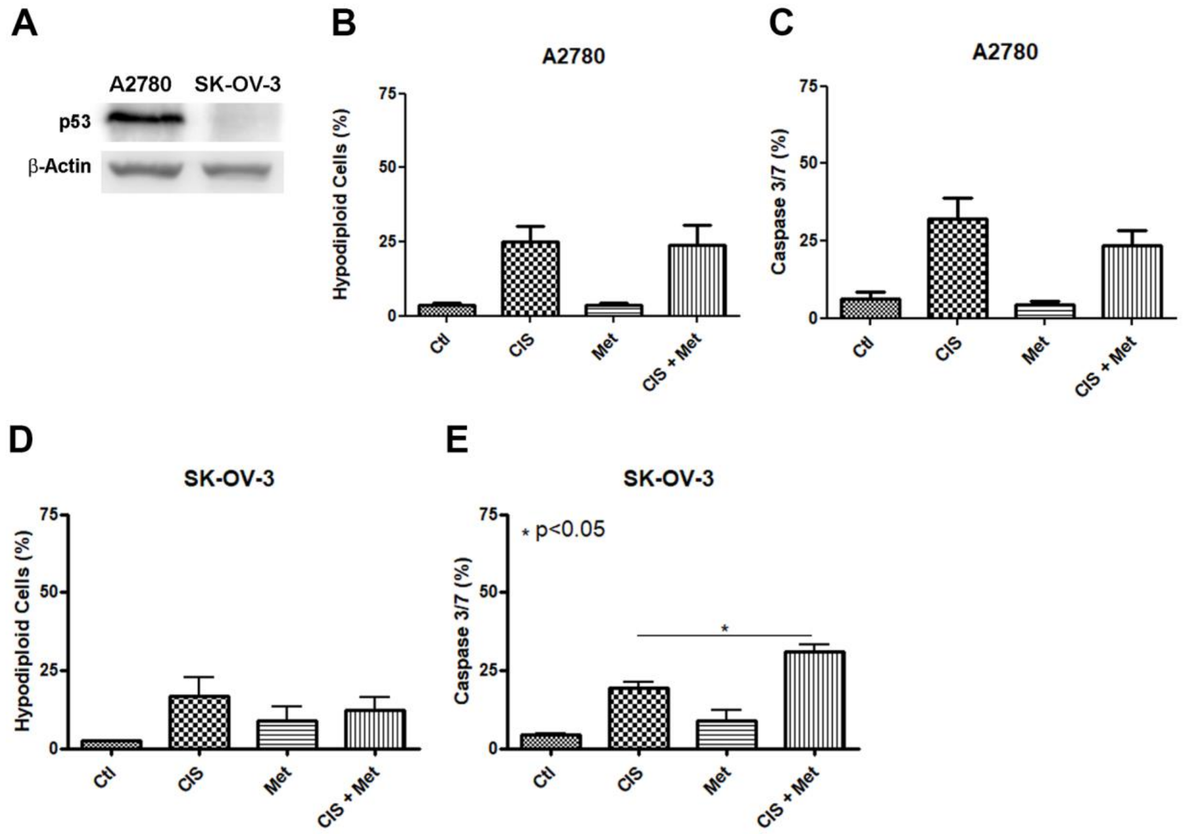

Supplementary Figure 5. Combination of metformin and cisplatin treatment in human ovarian carcinoma cells. Western blot showed that P53 is expressed in A2780 cells but not in SK-OV-3 cells (**A**). Combination of metformin and cisplatin treatment does not increase cell death, measured by hypodiploid cells and Caspase 3 and 7 activation in A2780 cells (**B**, **C**, respectively) and did not increased cell death by hypodiploid cells analysis in SK-OV-3 cells (**D**) but increased caspase 3 and 7 activation ($p<0.05$) (**E**). DNA fragmentation and Caspase 3 and 7 activation assays were performed according to materials and methods. Data represent the mean of three independent experiments. SK-OV-3 cells were treated with 20 mM of metformin for 72 h prior to the treatment with cisplatin (4 μM), combined or not with metformin, for another 72 h. A2780 cells were treated with 5 mM of metformin for 72 h prior to the treatment with cisplatin (2.5 μM), combined or not with metformin, for another 72 h.

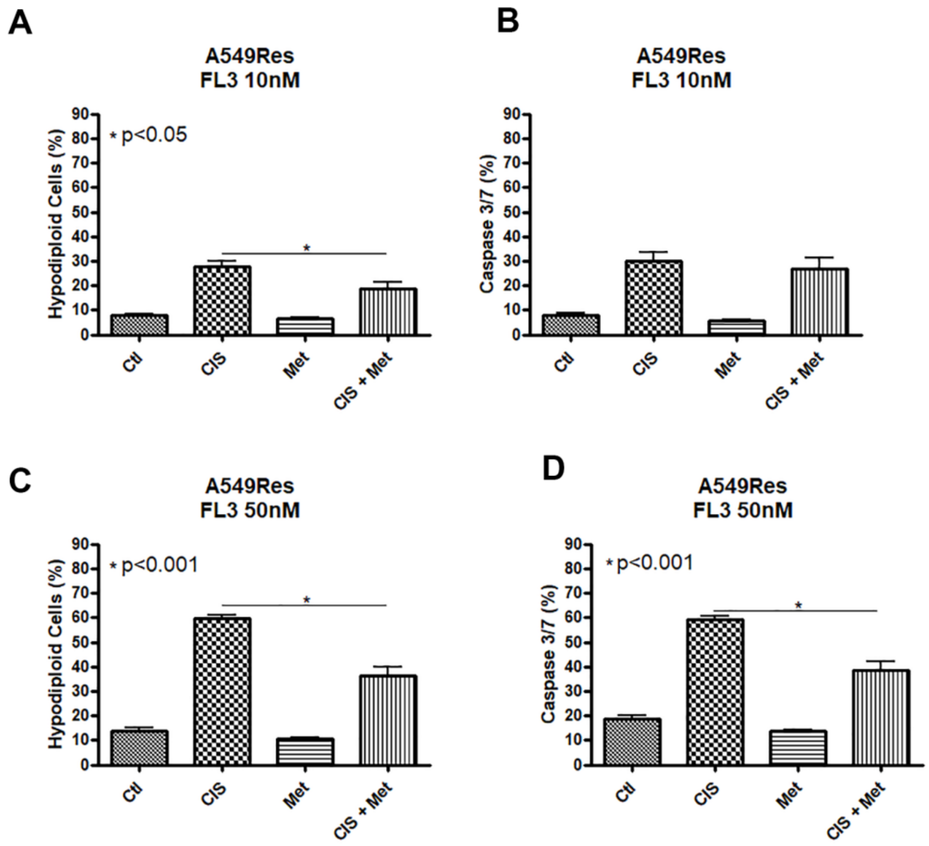

Supplementary Figure 6. FL3 protect A549Res cells to metformin and cisplatin combination. A549Res cells were treated with 10nM or 50nM of FL3 and then treated with the combination of metformin and cisplatin. Treatment with 10nM of FL3 protected the A549Res cells from cisplatin and metformin combination as seen by decreased DNA fragmentation ($p<0.05$) but did not change caspase 3 and 7 activation (**A**, **B**, respectively). When cells were treated with 50nM of FL3, the combination of cisplatin and metformin treatment decreased DNA fragmentation ($p<0.001$) and caspase 3 and 7 activation ($p<0.001$), when compared to cisplatin treatment alone, protecting A549Res cells (**C**, **D**, respectively). Sub-lethal treatment with cisplatin for generation of A549Res cells, treatment with FL3, cisplatin and metformin were made according to materials and methods. Data represent the mean of three independent experiments.

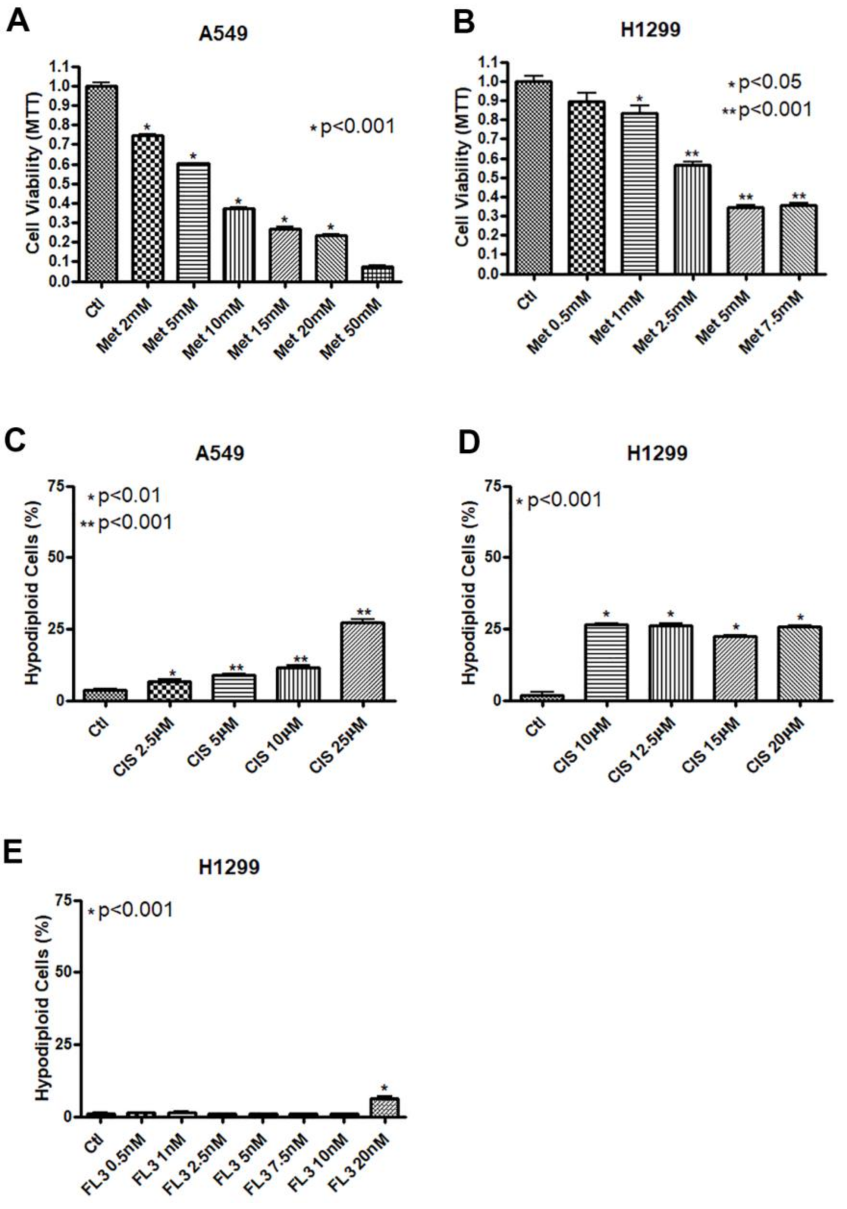

Supplementary Figure 7. Dose-response curves in A549 and H1299 cells for metformin, cisplatin and FL3. A549 and H1299 cells have different sensitivity to metformin and cisplatin. Cell viability for metformin treatment indicated that A549 cells are more resistant than H1299 cells (**A**, **B**). A549 cells were more resistant to cisplatin than H1299 cells (**C**, **D**). The smallest dose of FL3 before the induction of cell death (10nM), and a dose 5x higher (50 nM), was determined using the H1299 cells (**E**), and these concentrations were applied in the A549 cells. FL3, MTT and DNA fragmentation assay was made according to materials and methods. P value (*) or (**) is related to control (Ctl) group.

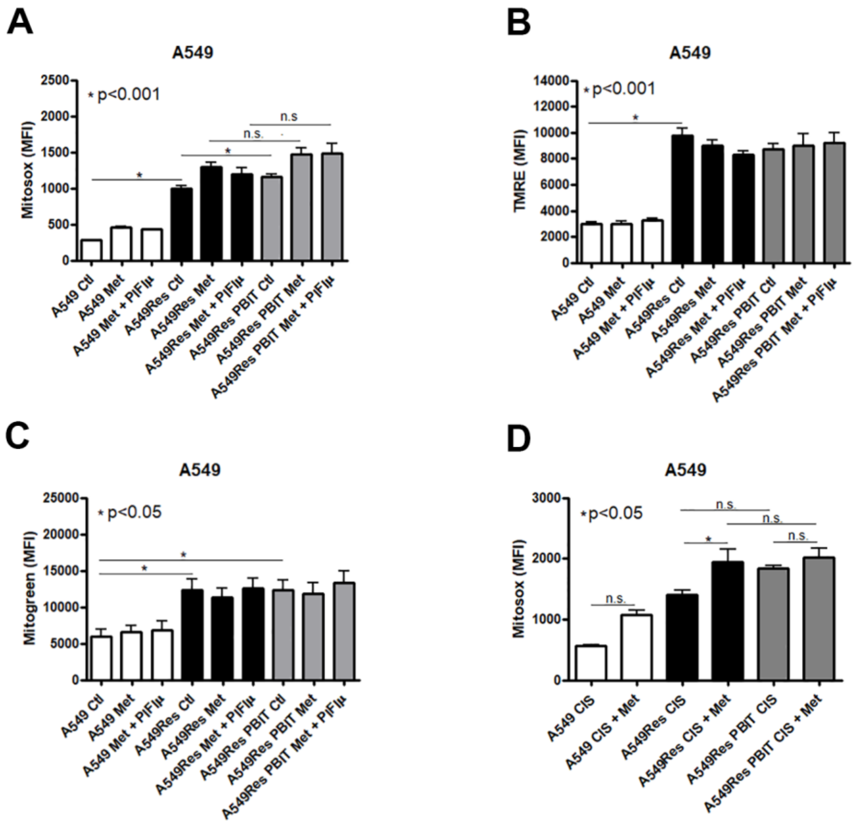

Supplementary Figure 8. Mitosox, TMRE and mitogreen analyses In A549 cells. Mitochondrial function was analyzed in A549 cells and in A549Res cells. Reactive oxygen species (ROS) increase was related to the sub-lethal treatment with cisplatin ($p<0.001$). PBIT treatment increased ROS levels, when compared to A549Res cells ($p<0.001$) but PBIT did not change ROS levels when cells were treated with metformin, even in the presence of pifithrin-μ (**A**). Mitochondrial membrane potential ($p<0.001$) and mitochondrial mass ($P<0.001$) increase was also related to the sub-lethal treatment with cisplatin and not to metformin treatment or by Jarid1b (**B**, **C**). Combined treatment with cisplatin and metformin increased ROS levels in A549Res cells ($p<0.05$) only when compared to cisplatin treatment alone, and PBIT treatment did no change ROS levels when compared to A549Res cells. (**D**). Sub-lethal treatment with cisplatin, combined treatment with cisplatin and metformin and Jarid1b inhibition by PBIT was made according to materials and methods. Non-significance (n.s.). Data represent the mean of three independent experiments.
